# Electromyography-Based Respiratory Onset Detection in COPD Patients on Non-Invasive Mechanical Ventilation

**DOI:** 10.3390/e21030258

**Published:** 2019-03-07

**Authors:** Leonardo Sarlabous, Luis Estrada, Ana Cerezo-Hernández, Sietske V. D. Leest, Abel Torres, Raimon Jané, Marieke Duiverman, Ainara Garde

**Affiliations:** 1 Biomedical Signal Processing and Interpretation, Institute for Bioengineering of Catalonia (IBEC), The Barcelona Institute of Science and Technology (BIST), 08028 Barcelona, Spain; 2 Department of Automatic Control (ESAII), Universitat Politècnica de Catalunya (UPC)—Barcelona Tech, 08028 Barcelona, Spain; 3 Biomedical Research Networking Center in Bioengineering, Biomaterials and Nanomedicine (CIBER-BBN), 08028 Barcelona, Spain; 4 Department of Pulmonology, Rio Hortega University Hospital, 47012 Valladolid, Spain; 5 Department of Pulmonary Diseases/Home mechanical Ventilation, University of Groningen, University Medical Center Groningen, 9713 Groningen, The Netherlands; 6 Biomedical Signals and Systems Group, Faculty of Electrical Engineering, Mathematics & Computer Science, University of Twente, 7500 Enschede, The Netherlands; 7 Groningen Research Institute of Asthma and COPD (GRIAC), University of Groningen, 9712 Groningen, The Netherlands

**Keywords:** fixed sample entropy, adaptive filtering, root mean square, diaphragm electromyography, non-invasive mechanical ventilation, chronic obstructive pulmonary disease

## Abstract

To optimize long-term nocturnal non-invasive ventilation in patients with chronic obstructive pulmonary disease, surface diaphragm electromyography (EMGdi) might be helpful to detect patient-ventilator asynchrony. However, visual analysis is labor-intensive and EMGdi is heavily corrupted by electrocardiographic (ECG) activity. Therefore, we developed an automatic method to detect inspiratory onset from EMGdi envelope using fixed sample entropy (fSE) and a dynamic threshold based on kernel density estimation (KDE). Moreover, we combined fSE with adaptive filtering techniques to reduce ECG interference and improve onset detection. The performance of EMGdi envelopes extracted by applying fSE and fSE with adaptive filtering was compared to the root mean square (RMS)-based envelope provided by the EMG acquisition device. Automatic onset detection accuracy, using these three envelopes, was evaluated through the root mean square error (RMSE) between the automatic and mean visual onsets (made by two observers). The fSE-based method provided lower RMSE, which was reduced from 298 ms to 264 ms when combined with adaptive filtering, compared to 301 ms provided by the RMS-based method. The RMSE was negatively correlated with the proposed EMGdi quality indices. Following further validation, fSE with KDE, combined with adaptive filtering when dealing with low quality EMGdi, indicates promise for detecting the neural onset of respiratory drive.

## 1. Introduction

Chronic obstructive pulmonary disease (COPD) is the fourth leading cause of death in the world [1,2]. Due to ageing of the population and continuation of risk factors, this problem will keep increasing in the coming decades. The disease is characterized by an airway obstruction leading to airflow limitation and, as a consequence, persisted respiratory symptoms such as dyspnea and production of sputum [1]. Patients with end-stage COPD frequently develop chronic hypercapnic respiratory failure, which is associated with end-of-life [3]. Long-term application of nocturnal intermittent non-invasive ventilation (NIV) in patients with chronic hypercapnic respiratory failure due to neuromuscular and thoracic restrictive disorders improves clinical outcomes and survival. However, this therapy has long been controversial in COPD patients [3,4]. With the introduction of high-intensity NIV, benefits of long-term NIV have been shown in stable COPD patients with chronic hypercapnic respiratory failure [3,5,6]. High-intensity NIV is defined as a mode of ventilation that delivers sufficient inspiratory positive airway pressure in combination with a higher backup breathing frequency to decrease arterial carbon dioxide levels. However, for some patients, adapting to high-intensity NIV is difficult and compliance rates in clinical practice are therefore sometimes disappointing. Furthermore, the response to NIV in terms of improvement in gas exchange and patient-centered outcomes such as improvement in health-related quality of life is variable between patients, despite the application of high-intensity NIV. A reason for a more prolonged adaption period, lower compliance rates and less effective ventilation might be the occurrence of patient-ventilator asynchrony with high-intensity NIV [7,8].

The surface diaphragm electromyography (EMGdi) signal provides a real-time indirect measure of neural respiratory drive, which reflects the load on the respiratory muscles [9]. Its non-invasive nature makes the method extremely useful during NIV; both to visually detect patient-ventilator asynchrony, and for repeated measurements [3]. However, for longer recordings, such as whole night recordings, visual inspection is burdensome and time-consuming. In addition, NIV is normally applied during sleep. Thus, there is a clear need to develop an automatic method to reliably detect the respiratory onset from EMGdi signal. This is crucial since the timing of respiratory effort versus the timing of the ventilator pressure wave determines the control of ventilation.

Nevertheless, the EMGdi signal is heavily contaminated by electrocardiographic (ECG) activity, compromising inspiratory onset detection. A number of methods have been used to estimate the envelope of EMGdi and automatically identify inspiratory onset [10,11,12,13]. Traditionally, the EMGdi envelope has been estimated using the average rectified value or the root mean square (RMS) of EMGdi signal after suppressing all QRS complexes (ECG interference) [11]. Recently, fixed sample entropy (fSE) has proved to be a more robust technique to estimate the amplitude variation in respiratory EMGdi signals. In addition, fSE permits extracting the EMGdi envelope without requiring a prior removal of QRS complexes [12,13]. However, poor EMGdi signal quality or high ECG interference can reduce the robustness of fSE. The use of adaptive filters has also been proposed to remove ECG interference and then estimate EMGdi amplitude [10]. However, these methods have been evaluated separately and in different contexts. Therefore, there is a clear need to explore whether reducing ECG interference by adaptive filtering can improve EMGdi envelope estimation and consequently respiratory onset detection.

In this study, we aim to compare the performance of previously proposed methods estimating EMGdi envelope and respiratory onset, to optimize the automatic detection of the onset of neural respiratory drive in COPD patients initiated on home NIV. First, to estimate EMGdi envelope, the use of fSE in combination with adaptive filtering was explored and compared to the RMS-based EMGdi envelope provided by the EMG acquisition device. Then, a dynamic threshold based on kernel density estimation (KDE), applied to the EMGdi envelopes, was proposed to detect the respiratory onset. The performance of the onset detection was validated using EMG-based visual scores completed by two well-trained clinicians.

## 2. Dataset and Visual Scores

### 2.1. Study Population

The clinical study was performed at the Department of Pulmonary Diseases of the University Medical Center Groningen (Groningen, The Netherlands). Nine patients with COPD initiated on home NIV in the hospital were included in this analysis. All patients participated in a randomized controlled trial of which investigating respiratory EMG and patient-ventilator asynchrony during NIV initiation was a secondary objective. Both studies, including the EMG measurements, were approved by the Ethical Committee Groningen (ClinicalTrials.gov NCT02652559 and NCT03053973). All patients gave written informed consent for participation in the trial and for the EMG measurements. Table 1 summarizes the characteristics and clinical data of the analyzed patients.

### 2.2. Data Acquisition

The EMGdi was measured in the COPD patients once they got used to sleeping with NIV during the night. In 7 patients nocturnal measurements were performed during the initiation period performed in the hospital after 2–5 nights NIV acclimatization and titration, and in 2 patients the EMG measurements were performed after a longer period of NIV used at home (in 1 patient a nocturnal measurement was performed after 6 months NIV use and in the other patient a daytime measurement was performed after more than 2 years NIV use). The EMGdi was measured using two 24 mm surface electrodes (Covidien Kendall Disposable Surface EMG/ECG/EKG Electrodes, Germany) placed bilaterally midclavicular at the costal margin. A common electrode was placed on the sternum. Moreover, a pressure sensor was placed between the mask and the ventilator to measure the pressure changes applied by the ventilator (DEMCON—Macawi Respiratory Systems BV, Enschede, The Netherlands). Both pressure and EMGdi signals were amplified and transferred to a laptop by the Dipha-16^®^ (DEMCON—Macawi Respiratory Systems BV, Enschede, The Netherlands). Data were recorded using Polybench data-acquisition and processing package (Applied Biosignals GMBH, Weener, Germany), with a sample frequency of 500 Hz [3].

### 2.3. Visual EMG Onset Detection

The data was transferred to MATLAB (The Mathworks, Inc., vR2014a, Natick, MA, USA) in order to perform the visual analysis and automatic onset detection. EMGdi signals were filtered with a 4th-order zero-phase Butterworth band-pass filter between 5 Hz and 400 Hz. Power line interference at 50 Hz and its harmonics were removed using a 10th-order zero-phase notch filters. For each patient, one hour of the EMGdi recording was selected. The EMGdi signals were visually evaluated separately by two physicians. The visual analysis included the onset annotations of every inspiration. A graphic user interface developed in MATLAB facilitated visually determining the onset points over the EMGdi signals (see Figure S1). The onset of diaphragm activity was selected as the point at which the surface EMGdi amplitude exceeds the value of basal expiratory activity. Respiratory cycles where the visual onset marks significantly differed between both experts (a difference greater than a specific tolerance value) were considered unreliable and removed from further analysis. The physicians, based on their clinical experience, set this tolerance value to 150 ms. When the visual onset marks were within the tolerance value, the mean of the two annotations was considered as the gold standard. This metric provides a more objective onset value that reduces the subjective judgments of single scorers, thus minimizing the imperfect nature of visual inspection scorers.

## 3. Data Analysis

An adaptive filter was applied to remove the cardiac interference from the EMGdi and improve signal quality for respiratory onset detection. Three EMGdi envelopes were used to automatically estimate the respiratory onset: the envelopes estimated applying fSE to both EMGdi and adaptively filtered EMGdi signals, and the envelope provided by the EMG acquisition device, which is based on the RMS calculation of the QRS-removed EMGdi signal. The performance between each method and the visual scorer reference onset value was evaluated through the root mean square error (RMSE). This metric is indifferent to the direction of errors. Therefore, the difference between visual score and automatic onset detection was taken to evaluate premature or delayed onset detections. In addition, the relationship between the EMGdi signal quality indexes and RMSE was assessed using the Pearson correlation coefficient (R). A single average value of EMG signal quality indexes and RMSE for each patient was used to calculate the R values. *p*-values lower than 0.05 were considered significant.

### 3.1. Quantifying EMGdi Signal Quality

The quality of EMGdi was evaluated by estimating the inspiratory activity increment with respect to basal expiratory activity, and the influence of the ECG interference [16]. The ratio between the mean power spectral density (PSD) of inspiratory segments without ECG and the mean PSD of expiratory segments without ECG (Rinex) was used as an expression of the increment of inspiratory EMGdi activity with respect to basal expiratory EMGdi activity. The higher the Rinex, the greater the difference between EMGdi inspiratory and expiratory segments. This ratio could be related to the ability of the automatic method to detect the inspiratory onset. In addition, the ratio between the mean PSD of inspiratory EMGdi segments without ECG and the mean PSD of expiratory EMGdi segments with ECG was used as an expression of the ECG interference over the EMGdi signals (Rcardio). The higher the Rcardio, the greater the difference between the amplitude of the ECG interference with respect to the inspiratory EMGdi.

**Figure 1:**
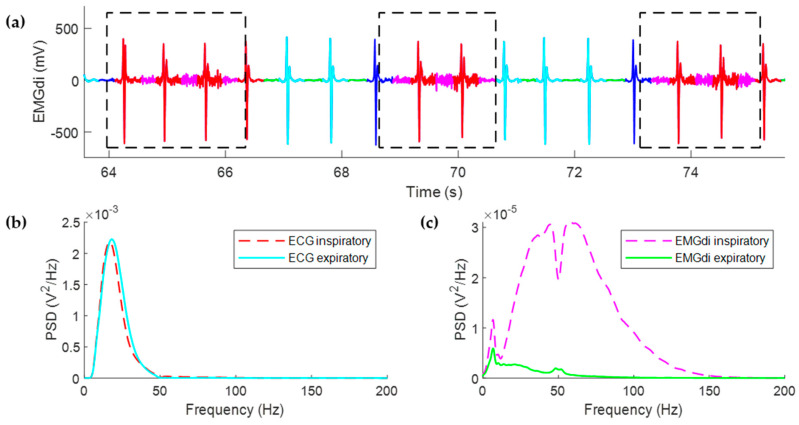
(**a**) EMGdi signal segments from a COPD patient with high quality signal recorded during non-invasive ventilation at night. Inspiratory cycles are representing by dotted line boxes. EMGdi inspiratory and expiratory segments without ECG interference (solid magenta and green traces, respectively), and ECG inspiratory and expiratory segments (solid red and cyan traces, respectively) are also shown. Solid blue traces shown not included segments. (**b**) PSD corresponding to ECG inspiratory and expiratory and (**c**) PSD corresponding to EMGdi inspiratory and expiratory phases.

Figure 1 depicts the different segments of EMGdi and the corresponding PSD. Inspiratory and expiratory EMGdi segments without ECG interference were selected, inside the inspiratory and expiratory cycle, respectively, as the EMGdi activity between the end of the T wave and the start of the P wave of the next beat. The ECG signal corresponds to a filtered EMGdi signal version between 0.5 Hz and 35 Hz with a zero-phase 4th order Butterworth filter. The PSD was calculated for each type of signal segment using the modified periodogram with a Hamming window and the discrete Fourier transform calculated with 4096 points, as described in reference [16].

### 3.2. Adaptive Filtering

**Figure 2:**
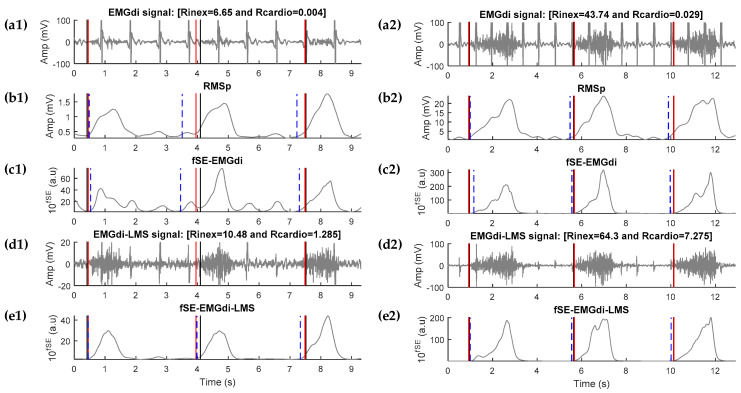
Representative examples of EMGdi signal (**a1**,**a2**) recorded in two patients with low (left panels) and high (right panels) signal quality, respectively. Derived root mean square (**b1**,**b2**) envelopes given by the EMG recording system (RMSp). Derived fixed sample entropy (fSE) envelopes over the EMGdi signal (fSE-EMGdi) (**c1**,**c2**). fSE was calculated using *m* = 1, *r* = 0.2 × standard deviation of EMGdi free of electrocardiographic interference and overlapping sliding window of 0.25 s. The EMGdi signal was adaptively filtered (**d1**,**d2**) using an LMS-based adaptive algorithm (EMGdi-LMS) and fSE (**e1**,**e2**) was also calculated (fSE-EMGdi-LMS). Rinex is an expression of increment of inspiratory EMGdi activity with respect to basal expiratory EMGdi activity, while Rcardio is an expression of the ECG interference over the EMGdi signals. Expert onset detections are shown in black solid lines (black and red lines). The onsets of neural respiratory activity (blue dotted lines) were detected through a dynamic threshold over the fSE-EMGdi and fSE-EMGdi-LMS using the Gaussian kernel density estimation approach.

We implemented an adaptive noise canceler based on event-synchronous cancellation to improve the quality of EMGdi signals by reducing the ECG interference. The adaptive noise canceler scheme used was previously proposed by references [17,18,19]. The EMGdi signal was used as the primary input to adaptive noise canceler. This signal, band pass filtered between 2 and 40 Hz, was used to detect the QRS complex. The reference input, uncorrelated with the EMGdi but correlated with the ECG interference, was artificially generated according to reference [19]. The number of weights of the linear combiner and the adjustment or adaptive constant of the Least-Mean-Square (LMS) algorithm were set to 10 and 10^−6^, respectively. That constant of adaptation minimized the energy difference between the output or cancellation signal and the reference input signal. The EMGdi-LMS signal represents the EMGdi signal adaptively filtered using the LMS algorithm (Figure 2(d1,d2)), and fSE-EMGdi-LMS represents the envelope extracted with fSE (Figure 2(e1,e2)).

### 3.3. EMGdi Envelope Estimation

#### 3.3.1. RMS-Based Method

The EMGdi envelope provided by the EMG acquisition device was based on the RMS method (RMSp) proposed by Prechtl et al. [11]. This method removes the electrical heart activity from the raw EMGdi by means of the gating technique. As was described by Prechtl, the QRS complexes are stretched into a standard QRS pulse and cut out of the raw EMGdi signal. The remaining EMGdi signal is averaged with a moving window and the RMS is calculated (Figure 2(b1, b2)) [11].

#### 3.3.2. Fixed Sample Entropy

Sample entropy (SampEn) is a measure of irregularity used to analyze short and noisy physiological time series [20,21]. It represents the conditional probability that two sequences of patterns of m consecutive samples, which are similar to each other within a tolerance *r*, will remain similar when one consecutive sample is added (*m* + 1). SampEn algorithm improves the performance of the approximate entropy [22] overcoming the bias due to self-matching counted and the lack of relative consistency [20] by the following procedure:

Given a time series of *N* samples x(n)=[x(1),x(2),…,x(N)], a subset of *N* − *m* + 1 overlapping vectors $X_{m}\left(i\right)$ of length *m* are constructed

(1) $$X_{m}\left(i\right)=\left[x\left(i\right),\;x\left(i+1\right),\ldots ,x\left(i+m-1\right)\right],\;\;i=1,\;2,\ldots ,N-m+1.$$

Then, define Chebyshev distance, i.e., the maximum absolute difference between corresponding pairs of scalar components of *m*-dimension vectors $X_{m}\left(i\right)$ and $X_{m}\left(j\right)$.

(2) $$d\left[X_{m}\left(i\right),X_{m}\left(j\right)\right]=\underset{k=0,\ldots ,m-1}{\max}\left[\left|x\left(i+k\right)-x\left(j+k\right)\right|\right],\;\;i\neq j.$$

$B_{i}\left(r\right)$ denotes the number of $X_{m}\left(j\right),\;j=1,\ldots ,\;N-m,\;i\neq j$ that resemble $X_{m}\left(i\right)$ and are less than or equal to a threshold *r*, such that $d_{m}\left[X_{m}\left(i\right),X_{m}\left(j\right)\right]\leq r$,  i≠j.

Then, for i = 1, 2,…N−m

(3) $$B_{i}^{m}\left(r\right)=\frac{B_{i}\left(r\right)}{N-m-1}.$$

The average for $B_{i}^{m}\left(r\right)$ es defined as

(4) $$B^{m}\left(r\right)=\frac{1}{N-m}\overset{N-m}{\underset{i=1}{\sum }}B_{i}^{m}\left(r\right).$$

This previous procedure is repeated, increasing the dimension *m* to *m* + 1 to obtain

(5) $$A^{m}\left(r\right)=\frac{1}{N-m}\overset{N-m}{\underset{i=1}{\sum }}A_{i}^{m}\left(r\right).$$

Thus, $B^{m}\left(r\right)$ is the probability that two sequences will match for *m* samples, whereas $A^{m}\left(r\right)\;$ is the probability that two sequences will match for *m* + 1 samples.

Sample entropy is then defined as

(6) $$\mathrm{SampEn}\left(m,r\right)=\underset{n\to \infty }{\lim}\left\{-ln\left[\frac{A^{m}\left(r\right)}{B^{m}\left(r\right)}\right]\right\},$$

which is estimated by the statistic:

(7) $$\mathrm{SampEn}\left(m,r,N\right)=ln\left[\frac{A^{m}\left(r\right)}{B^{m}\left(r\right)}\right].$$

The *m* parameter is generally taken as 2 while *r* parameter is set in range of 0.1 to 0.25 times the standard deviation of the segment analyzed of length *N* [23].

The fixed sample entropy (fSE), is a metric based on the original SampEn formulation to quantify the amplitude variation of complex components of stochastic signals [12,24]. fSE is calculated using a sliding window with overlap. fSE calculation requires the adjustment of three parameters: the embedding dimension *m*, the tolerance value *r* and the size of the sliding window (*N* parameter) [24,25]. The main difference between SampEn and fSE is that the latter uses a fixed tolerance value (*r*) defined as a fraction of the global standard deviation of the signal instead of the local standard deviation.

To optimize the neural respiratory activity estimation and reduction of ECG interference over EMGdi signal, the influence of several *m*, *r* and *N* input parameters were investigated in reference [25]. In the present study, based on recommendations of [25], fSE was explored using *m* equal to 1 and 2, *r* equal to 0.1, 0.2 and 0.3 times the standard deviation of the EMGdi signal and a sliding window equal to 0.25, 0.3, 0.4 and 0.5 s with 90% of overlap. Unlike in reference [25], the standard deviation of the EMGdi signal was calculated excluding ECG interference segments. The fSE parameters that provided the lowest geometric mean value of the RMSE were selected as the optimal parameter set.

To compute fSE-based EMGdi envelope we also applied a smoothing filter (using a moving average filter of 300 ms) and computed the exponential value of the resulting signal, to enhance high amplitude variations and reduce small amplitude variations, which might optimize onset detection (Figure 2(c1,c2)).

### 3.4. Automatic Respiratory Onset Detection

Breathing exhibits nonlinear patterns due to intrinsic changes associated to the respiratory system dynamic. Static thresholding methods used to detect onsets in EMGdi signals are limited since they provide only temporal information using the baseline amplitude characteristics of electromyographic signals [26]. Moreover, they can be affected by baseline variation such as ECG, movements artefacts or muscle interferences [26]. Thus, we propose the use of a dynamic thresholding that takes into account the breath by breath respiratory muscle variability [13]. This dynamic thresholding is based on the KDE, a nonparametric method used for the estimation of the probability density function *p*(*x*). KDE is calculated between two successive inspiratory cycles over the EMGdi envelope, which provides a smooth amplitude histogram. KDE estimation of the *p*(*x*) is given by:

(8) $$\hat{p}\left(x\right)=\frac{1}{Nh}\overset{N}{\underset{i=1}{\sum }}K\left(\frac{x-x_{i}}{h}\right),$$

where *K*(*x*) is the kernel function, *N* is the number of samples and *h* is the bandwidth or the smoothing parameter. We adopt a Gaussian kernel function of the form

(9) $$K\left(x\right)=\frac{1}{\sqrt{2\pi }}e^{-\frac{x^{2}}{2}}.$$

Assuming that the distribution is Gaussian and using a Gaussian kernel, the optimal bandwidth *h* is defined by (9), where σ is the standard deviation of the data [27]

(10) $$h_{\mathrm{opt}}=\sigma \sqrt[{5}]{\frac{4}{3N}}.$$

The dynamic threshold for each breathing was determined as the global maximum (mode) of the *p*(*x*). Finally, the inspiratory onset was then defined as the last point the threshold crosses the EMGdi envelope in upward direction. Figure 2 shows an example of automatic onset detection, with the different envelope extraction methods, for a patient with high signal quality (left panels) and a patient with low signal quality (right panels).

## 4. Results

### 4.1. Visual Onset Analysis

The scorers manually annotated 8117 breaths using the EMGdi as a reference. The cycles where the difference between scorers was higher than 150 ms were considered unreliable and excluded from further analysis (Table 2). The onset difference in ms and percentage of discarded cycles are represented in Table 2. On average, 26.5% of the cycles were rejected. Moderate negative correlations were observed between the percentage of discarded cycles and both Rinex (R = −0.63) and Rcardio (R = −0.43) indices.

### 4.2. EMGdi Signal Quality Analysis

In general, the EMGdi quality (Table 3) was varying, with some patients having lots of disturbance and others (i.e., patient 9) with high quality EMGdi signals, which was reflected by much lower errors detecting inspiratory onset. At large, both the Rinex and Rcardio indices improved after the adaptive filter, improving the EMGdi signal quality for monitoring respiratory drive. However, the ECG interference has not been reduced completely by the LMS approach as shown in Figure 2, leading in one case to the addition of noise to the filtered signal slightly reducing the Rinex index (i.e., patient 7, from 3.65 to 3.29).

### 4.3. RMSE Compared to Visual Analysis

**Figure 3:**
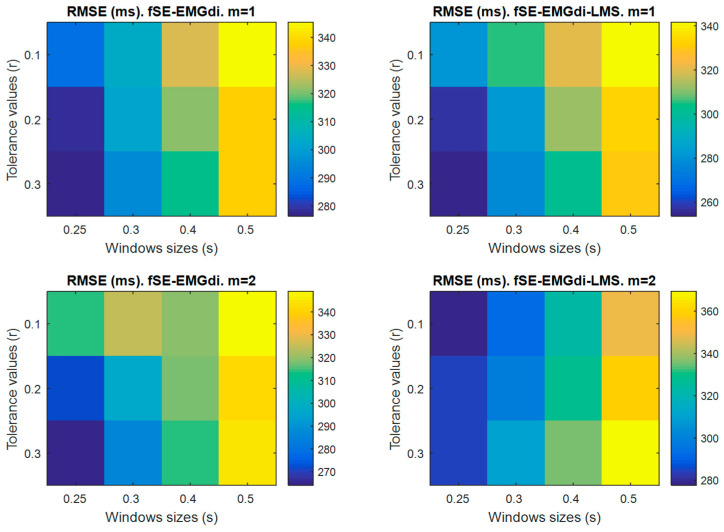
Root-mean-square error (RMSE) between the visual onset detection and automatic onset detections. Fixed sample entropy (fSE) was estimated over both the EMGdi signal (fSE-EMGdi) and filtered EMGdi signal using an LMS-based adaptive algorithm (fSE-EMGdi-LMS). fSE was calculated for different settings: *m* = 1 and 2, and tolerance values *r* = 0.1, 0.2 and 0.3 × standard deviation of EMGdi free of electrocardiographic interference, and overlapping sliding windows of 0.25, 0.3, 0.4 and 0.5 s. Each RMSE represents the geometric mean of obtained values for the 9 analyzed patients.

The geometric mean of the RMSE for all the analyzed patients, between gold standard onsets and automatic onsets estimated over the fSE envelopes using different settings is shown in Figure 3. The lower RMSE ranged between 253.7 and 341.6 ms for fSE-EMGdi-LMS (Figure 3). As illustrated, the parameter set that reduced the geometric mean RMSE detecting the respiratory onset using fSE-EMGdi (276.4 ms) and fSE-EMGdi-LMS (253.7 ms) was *m* = 1, *r* = 0.3, and sliding moving window equal to 0.25 s in both cases. Thus, this parameter set was considered optimal and was applied throughout the study.

The RMSE of each patient obtained between the different techniques and visual analysis is represented in Table 4. The lowest mean RMSE was observed for fSE-EMGdi-LMS, while the lowest RMSE dispersion was shown for RMSp followed by fSE-EMGdi-LMS. In general, RMSE decreases when the adaptive filter is applied. However, in some patients the RMSE obtained over fSE-EMGdi was lower than obtained by RMSp. The correlations between Rinex and Rcardio indexes (Table 3) and RMSE (Table 4) were assessed. Moderate and strong negative correlation were observed between Rinex and RMSE for fSE-EMGdi (R = −0.62) and fSE-EMGdi-LMS (R = −0.8, *p*-value < 0.05), respectively. Moderate and strong negative correlation were also observed between Rcardio and RMSE for fSE-EMGdi (R = −0.43) and fSE-EMGdi-LMS (R = −0.77, *p*-value = 0.015), respectively. The performance of the different techniques was also evaluated against the visual annotations carried out separately by the two physicians. However, the mean of the two annotations provided more reliable results than those obtained using a single observer (see Table S1).

### 4.4. Premature or Delayed Onset Detection Comparison

**Figure 4:**
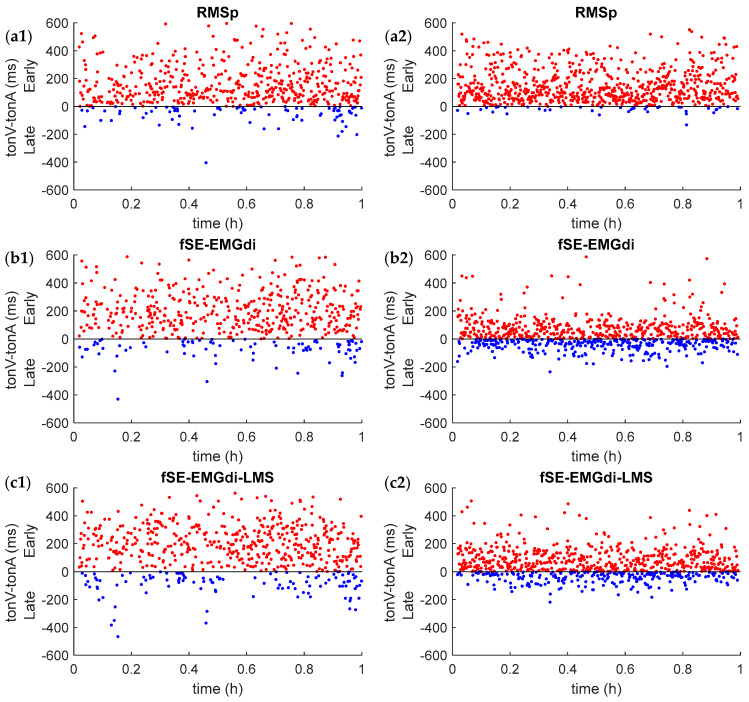
Comparison between the onset average detection of two visual scorers (tonV) and the automatic onset detection, including all studied methods, in two representative patients with low (left panels) and high (right panels) quality signal, respectively. Automatic detections were estimated from RMS-based (**a1**,**a2**) envelopes provided by the acquisition device (RMSp), fSE-based envelopes (**b1**,**b2**) over the EMGdi signal (fSE-EMGdi), and fSE-based envelopes (**c1**,**c2**) from the LMS-based adaptively filtered EMGdi signal (fSE-EMGdi-LMS). Upper red dots represent early detection and lower blue dots represent late detection of the automatic methods.

All automatic methods identified respiratory onset before the visual onset marks (average of both scorers). This is clearly reflected in Table 5, where the difference between the visual analysis and automatic onset detection is represented per patient for all studied methods. This effect is clearly illustrated in Figure 4, where the visual scores and automatic onset detection, with the different methods are compared, for a patient with high signal quality (left panels) and a patient with low signal quality (right panels). In both cases the onset was detected before the visual scores and the mean error and dispersion was clearly higher for the patient with lower EMGdi signal quality.

## 5. Discussion

In the present study, we developed a new approach to automatically detect respiratory onset from EMGdi. We proposed a novel method using sample entropy to compute the envelope of EMGdi and a Gaussian-KDE based threshold to non-invasively estimate the onset of the inspiratory activity in COPD patients initiated on home NIV in the hospital. The envelope, derived using fSE (mean RMSE = 298 ms), showed slightly higher values in estimating the onset compared to the RMS-based envelope (mean RMSE = 301 ms) provided by the EMG acquisition device; both evaluated during day and/or night, and with poor EMG signal-to-noise ratios.

In addition, this study is the first to our knowledge to introduce the combination of the fSE and the adaptive filtering techniques to improve the estimation of EMGdi envelope when dealing with low quality EMGdi signals, which further reduced the RMSE to 264 ms. Furthermore, we proposed EMGdi quality indexes to assess the impact of EMGdi signal quality detecting inspiratory onset. This showed that there are significant moderate to strong negative correlations between the quality indices and RMSE. However, as observed for patient 7, the LMS approach slightly reduced the Rinex index from 3.65 to 3.29, without compromising the performance of the automatic onset detections. All automated methods detected the onset before the visual scores; with a mean premature detection of 159 ms, 152 ms and 196 ms, for fSE-EMGdi, fSE-EMGdi-LMS and RMSp, respectively. The mean and dispersion of onset detection errors were higher for patients with lower EMGdi signal quality.

The estimation of inspiratory onset is the necessary key step to detect patient-ventilator asynchrony, which is normally done by visual inspection. In an attempt to automatize patient-ventilator asynchrony detection, we showed that it is feasible to automatically detect inspiratory onset from EMGdi. However, compared to visual analysis, the automatic methods estimate the onset prematurely with an error ranging from 30 ms to 309 ms, 47 ms to 233 ms, and 89 ms to 255 ms, using fSE-EMGdi, fSE-EMGdi-LMS and RMSp, respectively.

Nevertheless, it should be considered that the variability between visual scorers was very high. The lack of exact mathematical definition of the EMGdi onset represents a challenging problem [28]. This is reflected by the percentages of cycles removed, up to 45% in one patient with low EMGdi signal quality, because the inter-scorers difference was very high. Only cycles where the scorers agreed within a tolerance of 150 ms were included in the analysis. The mean between both scores was considered the reference onset, which included some error in our analysis, as both marks could be 150 ms apart. The main source of variability was the onsets that lay close to QRS complexes. In addition, the visual marks were performed in the EMGdi signal, without the adaptive filter that reduced ECG interference, which might have negatively influenced the visual onset detection. This can be observed in Figure 2, where the automatic marks look more reliable than the visual marks.

Several studies based on sample entropy analysis have properly estimated respiratory onset without removal of ECG artifacts from good quality EMGdi signals [13,29,30]. The main challenge on onset detection studies using the fSE technique is to determine the optimal parameters. Recommendations for estimating respiratory muscle activity using fSE on EMGdi for different levels of inspiratory effort developed by a healthy subject in seated position were established by Estrada et al. [25]. However, such recommendations were not made considering surface EMGdi signals from COPD patients with home mechanical ventilation. Therefore, we investigated the effect of fSE parameters: *r* values of 0.1, 0.2 and 0.3 times the standard deviation of the EMGdi signal excluding ECG interference segments, *m* = 1 and 2, and overlapping sliding windows between 0.25 s to 0.5 s on estimation of neural onset. The optimal parameters were set to *m* = 1, *r* = 0.3, and sliding moving window equal to 0.25 s, based on the lowest geometric mean RMSE found for fSE-EMGdi (276.4 ms) and fSE-EMGdi-LMS (253.7 ms). Interestingly, the selected parameters were in accordance with the recommendations made by Estrada et al. [25], and were suitable to extract respiratory muscle activity in COPD patients undergoing NIV.

Other problems related to background noise presented during EMG signal recording and signal to noise ratio notably could also affect the accuracy of onset detection [31]. In studies with EMGdi signals, the interference and frequency overlap between ECG and EMGdi signals can affect the analysis and interpretation of researchers and clinicians. ECG and EMGdi signals could be viewed as being derived from two dynamic systems with different complexity characteristics [29]. Despite fSE permits extracting the EMGdi envelope without removing ECG interference, the quality of EMGdi is a key factor in robust envelope estimation. Thus, in this study, for the first time, we proposed EMG quality indices and a combined adaptive filter with fSE to detect respiratory onset when dealing with challenging EMGdi signals. These quality indices enabled quantifying the performance of adaptive filtering. Although the ECG interference was not completely removed, the quality of the EMGdi signal improved, which enhanced the fSE performance and the automatic onset detection. Nevertheless, despite being beneficial in low quality signals, adaptive filtering is very time consuming and could be avoided when working with high quality signals since the improvement could be minimal.

Patient-ventilator interaction is generally evaluated through indirect estimates of neural onset time based on a drop in esophageal pressure and the onset of airflow at the mouth. However, indirect estimates of neural onset can be affected by physiological factors such as intrinsic positive end-expiratory pressure (PEEPi), or mechanical changes of rib cage [32]. Therefore, more direct measures of respiratory muscle electrical activity have been proposed to alleviate imprecisions whilst using surrogate estimates of neural onset in mechanical ventilation. Although it is possible to detect diaphragm EMG with esophageal or even needle electrodes, these invasive methods are impractical in patients on chronic home NIV. Therefore, we focused on using surface EMG, being aware of its drawbacks in terms of more signal noise and crosstalk by other muscles, to detect respiratory neural drive in patients on home mechanical ventilation.

The present study does have certain limitations. Firstly, it is a study with a relatively small sample size that could limit the generalization of the results. A second limitation is that despite the visual EMG onset detection approach being the gold standard and considered to provide accurate onset detection, this method strongly depends on the experience of the expert. It is highly subjective and with poor reproducibility, and thus could be heavily biased by personal skills [28]. A third limitation is the fact that the EMGdi signals, unlike EMG signals recorded in other muscles, have low signal-to-noise ratio and the manual annotations are more difficult to make. Therefore, in future works, we plan to make manual annotations over the EMGdi-LMS signals and compare them with those made on the EMGdi signal. Another limitation of the study is that the implemented adaptive noise canceler filter was based on a fixed ECG template. In the future, we will investigate whether generating an adaptive noise canceler that better represents the variability of the cardiac pattern, improves the cancelation of ECG interference. In this sense, different templates obtained every 5 min will be used instead of single template. We will also explore the use of other adaptive filtering algorithms such as the Recursive-Least-Squares algorithm.

## 6. Conclusions

In this work, a new fSE-based approach to detect neural onset from muscle respiratory signals was proposed for COPD patients during non-invasive ventilation. The performance of fSE was improved including an adaptive filtering step that allowed us to reduce cardiac interference when evaluating EMGdi recordings with low signal quality. The fSE combined with the KDE resulted in a suitable tool for onset detection where the muscle activation profile can be difficult to evaluate. Our findings suggest that using fSE is promising to detect neural onset from muscle respiratory signals in COPD patients during non-invasive ventilation. We recommend using fSE alongside adaptive filtering when EMGdi recordings have low signal-to-noise ratio. More research is required to further validate our findings in a larger cohort and to investigate whether it can be used to detect and treat patient-ventilator asynchrony in order to improve clinical outcomes.

## Figures and Tables

**Table 1:** Characteristics and clinical data of the included COPD patients.

Sex, female/male (n)	5/4
Age (years)	63 (58–72)
BMI (kg/m^2^)	25 (22–29)
FEV_1_ (L)	0.59 (0.52–0.75)
FEV_1_ (%pred)	20 (16–26)
FVC (L)	2.22 (1.43–2.63)
FVC (%pred)	53 (44–66)
FEV_1_/FVC (%)	28 (23–33)
TLC (L)	8.01 (6.82–9.22)
TLC (%pred)	134 (116–156)
RV (L)	5.29 (4.88–6.21)
RV (%pred)	252 (215–267)
RV/TLC (%)	66 (61–74)
PaCO_2_ before NIV initiation (kPa)	6.6 (6.2–8.9)
PaO_2_ before NIV initiation (kPa)	8.3 (5.9–9.4)

BMI, body mass index; FEV_1_, postbronchodilator forced expiratory volume in 1 s in liters (L); FVC, forced vital capacity in liters (L); RV, residual volume; TLC, total lung capacity; %pred: percentage of the predicted values [14,15]; PaO_2_, arterial oxygen pressure at daytime without ventilation; PaCO_2_, arterial carbon dioxide pressure at daytime without ventilation; kPa, kilopascal; NIV, non-invasive ventilation. Characteristics are represented by their median and inter-quartile range.

**Table 2:** Onset difference between manual detections considering reliable breaths less than 150 ms. Percentage of cycles removed.

COPD	Differences between Scores (ms)	Discarded Cycles (%)
1	−6 ± 77	34.2
2	−32 ± 73	39.3
3	15 ± 74	27.1
4	10 ± 68	21.0
5	−10 ± 70	40.0
6	−13 ± 67	24.4
7	6 ± 81	45.0
8	33 ± 56	5.4
9	16 ± 46	2.2
Mean ± SD	5 ± 69	26.5 ± 15.1

**Table 3:** Rinex and Rcardio indices calculated over the EMGdi signal before and after applying the LMS-based adaptive algorithm.

	Rinex		Rcardio	
COPD	EMGdi	EMGdi-LMS	EMGdi	EMGdi-LMS
1	1.70	1.73	0.0060	0.4019
2	5.09	6.59	0.0009	0.5051
3	6.31	6.57	0.0082	0.3306
4	3.44	4.82	0.0100	0.0790
5	6.65	10.48	0.0035	1.2849
6	9.63	15.18	0.0047	0.6149
7	3.65	3.29	0.0129	0.1493
8	5.82	9.23	0.0011	0.2751
9	43.74	64.30	0.0293	7.2753

EMGdi-LMS: EMGdi signal filtered using an LMS-based adaptive algorithm. Rinex: ratio between the mean power spectral density (PSD) of inspiratory segments without ECG and the mean PSD of expiratory segments without ECG. Rcardio: ratio between the mean PSD of inspiratory EMGdi segments without ECG and the mean PSD of expiratory EMGdi segments with ECG.

**Table 4:** The RMSE (in ms) of the automatic respiratory onset detection per patient.

COPD	fSE-EMGdi	fSE-EMGdi-LMS	RMSp
1	303	318	353
2	335	316	303
3	317	264	376
4	523	346	282
5	369	293	339
6	278	273	277
7	251	225	333
8	187	220	255
9	116	120	194
Mean ± SD	298 ± 115	264 ± 68	301 ± 56

fSE-EMGdi: fixed sample entropy (fSE) estimated over the EMGdi signal. fSE-EMGdi-LMS: fSE estimated over filtered EMGdi signal using an LMS-based adaptive algorithm. fSE was calculated using *m* = 1, tolerance values *r* = 0.3 × standard deviation of EMGdi free of electrocardiographic interference and overlapping sliding windows of 0.25 s. RMSp: root mean square given by the recording system.

**Table 5:** The difference (in ms) between the visual scores (average detection of two visual scorers) and the automatic onset detection per patient.

COPD	fSE-EMGdi	fSE-EMGdi-LMS	RMSp
1	101 ± 286	99 ± 302	220 ± 276
2	211 ± 260	233 ± 214	239 ± 186
3	157 ± 276	138 ± 226	223 ± 302
4	309 ± 422	204 ± 280	174 ± 222
5	222 ± 296	204 ± 210	255 ± 224
6	199 ± 194	180 ± 205	209 ± 181
7	80 ± 238	47 ± 221	89 ± 321
8	129 ± 136	163 ± 148	183 ± 177
9	30 ± 112	47 ± 110	139 ± 136
Mean ± SD	159 ± 261	152 ± 218	196 ± 221

fSE-EMGdi: fixed sample entropy (fSE) estimated over the EMGdi signal. fSE-EMGdi-LMS: fSE estimated over filtered EMGdi signal using an LMS-based adaptive algorithm. fSE was calculated using *m* = 1, tolerance values *r* = 0.3 × standard deviation of EMGdi free of electrocardiographic interference and overlapping sliding windows of 0.25 s. RMSp: root mean square given by the recording system.

## Supplementary Materials

The following are available online at https://www.mdpi.com/1099-4300/21/3/258/s1, Figure S1: Graphic user interface to determining the onset points, Table S1: The performance of the different techniques evaluated against the visual annotations carried out separately by the two physicians and the mean of the two annotations.
