# Supplementary material for: Electromyography-Based Respiratory Onset Detection in COPD Patients on Non-Invasive Mechanical Ventilation
Source: Entropy (Basel). 2019 Mar 7;21(3):258. doi: 10.3390/e21030258 (PMC7514739; doi:10.3390/e21030258)
Supplement: Supplementary file 1 [file entropy-21-00258-s001.zip › Table S1_AG.docx]

**Table S1: The performance of the different techniques evaluated against the visual annotations carried out separately by the two physicians and the mean of the two annotations.**

To analyse the subjective judgments of single scorers (scorer1 and scorer2), and to highlight the imperfect nature of the gold standard (scorerM, the mean of the two annotations), a comparison between automatic onset detection and scorer1, scorer2 and scorerM were performed. The root mean square error (RMSE) was used to evaluate the performance between each method and the visual scorer reference onset value. In the three cases, the lower RMSE was obtained using fSE-EMGdi-LMS, ranging between 256.7 and 343.7 ms, 255.5 and 343.1, and 253.7 and 341.6 ms for scorer1, scorer2 and scorerM, respectively. Furthermore, as presented in the original manuscript, the optimal set of parameters that reduced the geometric mean RMSE were *m*=1, *r*=0.3, and a sliding moving window of 0.25s.

Table 1 shows the RMSE using the optimal set of parameters for the different techniques and visual analysis. This table represents an extended version of Table 4 in the original manuscript but including scorer1 and scorer2. As it can be observed, the lower RMSE (for each patient) was not always obtained with the same scorer. The lowest mean RMSE was found when using the mean of the two annotations. Table 2 shows the direction of the error (premature or delayed detections) represented as the mean and the standard deviation between the visual analysis and automatic onset detection per patient for all studied methods. This table represents an extended version of Table 5 in the original manuscript but including scorer1 and scorer2. As it can be observed, the lower RMSE (for each patient) was not always obtained with the same scorer. Therefore, due to the intrinsic subjective difference between the two scorers, in this study, we have proposed to use the mean between both scorers as a more reliable reference of respiratory onset.

In conclusion, this metric provides a more objective onset value that reduces the subjective judgments of single scorers thus minimizing the imperfect nature of visual inspection scorers.

Table 1. Root mean square error between (in ms) of the automatic respiratory onset detection per patient.

|  | **fSE-EMGdi** | | | **fSE-EMGdi-LMS** | | | **RMSp** | | |
| --- | --- | --- | --- | --- | --- | --- | --- | --- | --- |
| **COPD** | scorer1 | scorer2 | Mean | Scorer1 | Scorer2 | Mean | Scorer1 | Scorer2 | Mean |
| 1 | 301 | 310 | 303 | 317 | 323 | 318 | 350 | 360 | 353 |
| 2 | 324 | 349 | 335 | 303 | 333 | 316 | 291 | 319 | 303 |
| 3 | 320 | 318 | 317 | 271 | 262 | 264 | 379 | 376 | 376 |
| 4 | 526 | 523 | 523 | 349 | 346 | 346 | 286 | 281 | 282 |
| 5 | 371 | 372 | 369 | 295 | 295 | 293 | 340 | 343 | 339 |
| 6 | 277 | 283 | 278 | 269 | 281 | 273 | 275 | 282 | 277 |
| 7 | 256 | 252 | 251 | 231 | 227 | 225 | 335 | 335 | 333 |
| 8 | 202 | 178 | 187 | 236 | 208 | 220 | 269 | 244 | 255 |
| 9 | 119 | 118 | 116 | 123 | 121 | 120 | 202 | 190 | 194 |
| Mean±SD | 300±113 | 300±116 | **298±115** | 266±65 | 266±72 | **264±68** | 303±54 | 303±60 | **301±56** |

fSE-EMGdi: fixed sample entropy (fSE) estimated over the EMGdi signal. fSE-EMGdi-LMS: fSE estimated over filtered EMGdi signal using an LMS-based adaptive algorithm. RMSp: root mean square of the EMGdi signal provided by de acquisition device.

Table 2. Difference (in ms) between automatic onset detection and each visual score (scorer 1 and scorer 2) and the average detection of two visual scorers per patient.

|  | | **fSE-EMGdi** | | | | **fSE-EMGdi-LMS** | | | | | | **RMSp** | | | | |
| --- | --- | --- | --- | --- | --- | --- | --- | --- | --- | --- | --- | --- | --- | --- | --- | --- |
| **COPD** | Scorer1 | | Scorer2 | Mean | Scorer1 | | Scorer2 | | | Mean | Scorer1 | | Scorer2 | Mean | |  |
| 1 | | 98±284 | 105±292 | 101±286 | | 96±303 | | 102±207 | 99±302 | | | 217±275 | 223±282 | | 220±276 | |
| 2 | | 195±260 | 227±266 | 211±260 | | 217±213 | | 249±221 | 233±214 | | | 223±187 | 255±192 | | 239±186 | |
| 3 | | 164±275 | 149±281 | 157±276 | | 146±229 | | 131±228 | 138±226 | | | 231±301 | 216±309 | | 223±302 | |
| 4 | | 314±422 | 304±426 | 309±422 | | 209±280 | | 198±284 | 204±280 | | | 179±223 | 169±225 | | 174±222 | |
| 5 | | 217±301 | 227±295 | 222±296 | | 199±218 | | 209±208 | 204±210 | | | 250±230 | 260±223 | | 255±224 | |
| 6 | | 193±200 | 206±194 | 199±194 | | 174±206 | | 187±210 | 180±205 | | | 203±186 | 216±182 | | 209±181 | |
| 7 | | 83±242 | 77±240 | 80±238 | | 50±226 | | 44±223 | 47±221 | | | 92±323 | 86±324 | | 89±321 | |
| 8 | | 145±140 | 112±138 | 129±136 | | 180±152 | | 146±148 | 163±148 | | | 200±180 | 166±179 | | 183±177 | |
| 9 | | 38±113 | 22±116 | 30±112 | | 56±110 | | 39±115 | 47±110 | | | 147±138 | 131±137 | | 139±136 | |
| Mean±SD | | 161±261 | 156±256 | 159±261 | | 155±219 | | 150±222 | 152±218 | | | 196±222 | 191±225 | | 196±221 | |

fSE-EMGdi: fixed sample entropy (fSE) estimated over the EMGdi signal. fSE-EMGdi-LMS: fSE estimated over filtered EMGdi signal using an LMS-based adaptive algorithm. fSE was calculated using m = 1, tolerance values r = 0.3 x standard deviation of EMGdi free of electrocardiographic interference and overlapping sliding windows of 0.25 s. RMSp: root mean square given by the recording system.
