# Supplementary material for: Electromyography-Based Respiratory Onset Detection in COPD Patients on Non-Invasive Mechanical Ventilation
Source: Entropy (Basel). 2019 Mar 7;21(3):258. doi: 10.3390/e21030258 (PMC7514739; doi:10.3390/e21030258)
Supplement: Supplementary file 1 [file entropy-21-00258-s001.zip › Figure S1.docx]

**Figure S1: Graphic user interface to determining the onset points.**

In order to determining the onset points over the surface EMGdi signals a graphic user interface was developed. This graphic user interface was developed using MATLAB (The Mathworks, Inc., vR2014a, Natick, MA, USA). Figure S1 shows a screenshot of graphic user interface. Onset and offset points are represented as red and green dotted vertical lines, respectively. The onset of diaphragm activity was selected as the point at which the surface EMGdi amplitude exceeds the value of basal expiratory activity.


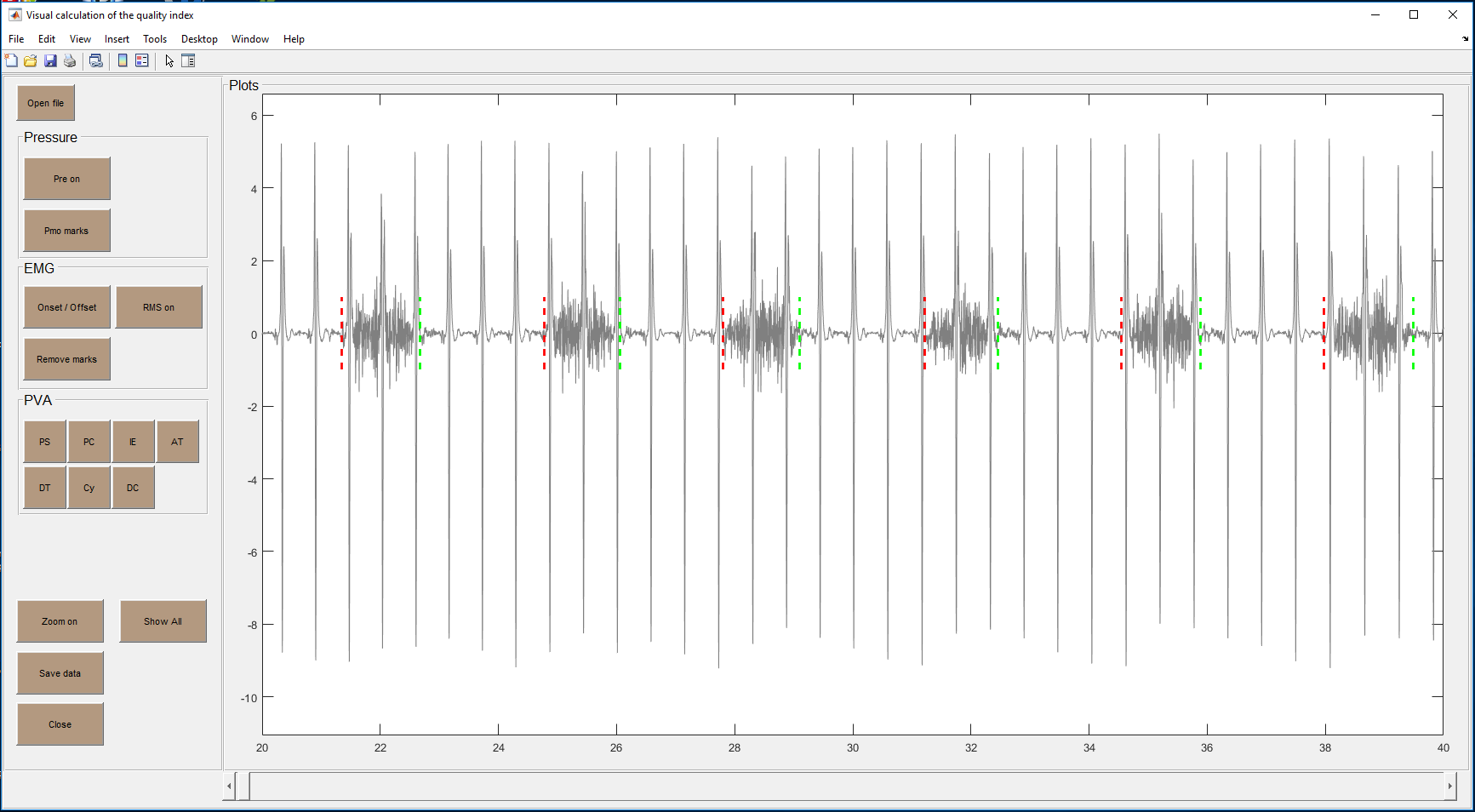


Figure S1: Graphic user interface to determining the onset points.
